# Quality of life among germ-cell testicular cancer survivors: The effect of time since cancer diagnosis

**DOI:** 10.1371/journal.pone.0258257

**Published:** 2021-10-06

**Authors:** Aleksandar Jovanovski, Daniela Zugna, Daniela Di Cuonzo, Patrizia Lista, Libero Ciuffreda, Franco Merletti, Rosalba Rosato, Lorenzo Richiardi

**Affiliations:** 1 Cancer Epidemiology Unit, Department of Medical Sciences, University of Turin and CPO-Piemonte, Torino, Italy; 2 Department of Clinical and Biological Sciences, University of Turin, Turin, Italy; 3 Medical Oncology Division 1, University Hospital "Citta’ della Salute e della Scienza", Turin, Italy; 4 Department of Psychology, University of Turin, Turin, Italy; Tabriz University of Medical Sciences, ISLAMIC REPUBLIC OF IRAN

## Abstract

**Introduction:**

Testicular cancer is one of the most treatable cancers, with a 10-year survival of more than 95%. Many patients will be long-term survivors and this disease strikes men in an important phase of their lives, therefore the quality of life (QoL) among these patients is an area of particular interest. We aimed to study whether QoL in testicular cancer survivors depends on the time since cancer diagnosis.

**Methods:**

Data were collected from the EPSAM (Esposizioni postnatali e salute maschile) study, a case-control study on patients with testicular cancer, diagnosed between 1997 and 2008 in the province of Turin, Northern Italy, and interviewed between 2008 and 2010 (response rate among cases **57%**). Patients were contacted through their oncologist at the San Giovanni Batista Hospital in Turin or through their general practitioner (GP) in the rest of the Province of Turin. QoL was assessed cross-sectionally using the short form 12 (SF-12) questionnaire, a generic short-form health survey that produces two summary scores, PCS (physical component score) and MCS (mental component score), to evaluate physical and mental health, respectively.

**Results:**

Out of **234** study patients, **125** cases were seminomas and **109** cases were nonseminomas. The mean age at diagnosis was **34.5** years. After adjusting for age, time since diagnosis was not associated with PCS and MCS scores. Among nonseminomas, the median PCS slightly increased (adjusted OR (odds ratio) for 5+ vs < 2 years since cancer diagnosis: **1.78 (1.17–2.73), p = 0.008**) and MCS slightly decreased (adjusted **OR** per 1-year increase since cancer diagnosis**: 0.92, 95% CI: 0.82–1.05, p = 0.23**) with time. Similar findings of no association between time since diagnosis and PCS and MCS were found when the analyses were restricted to the subgroup of cancer patients contacted through their oncologist, whose response proportion was 82%.

**Conclusion:**

In a study of testicular cancer patients interviewed cross-sectionally at 1 to more than 10 years since diagnosis, time since cancer diagnosis was not associated with QoL when we considered all germ-cell testicular cancer patients together. When stratified by histology type, we found certain evidence that nonseminoma cases report higher PCS over time since cancer diagnosis.

## Introduction

Testicular cancer represents 1–3% of all cancers among males [1–3]. Incidence peaks at around 30 years of age and testicular cancer is the most frequent tumor among young men [1–3]. Moreover, its incidence has been increasing over the last few decades in many populations [1]. Nowadays, testicular cancer is one of the most treatable cancers, with a 10-year survival rate of more than 95% [4]. This disease strikes men in an important phase of their lives, which is often characterized by the start of a career and/or a family [5]. Thanks to a successful treatment, testicular cancer patients will be long-term survivors, therefore their quality of life is an area of particular interest. Treatment for testicular cancer is based on three fundamental options: surgery, radiotherapy and chemotherapy. Cancer treatment may lead to considerable morbidity, including sexual and fertility dysfunction after resection of retroperitoneal lymphatic nodes [6]; renal, cardiovascular, neurological or pulmonary diseases after chemotherapy; and gastrointestinal problems after radiotherapy [7–12]. Both radiotherapy and chemotherapy increase the risk of second primary cancers [13]. Previous studies about quality of life among testicular cancer survivors suggest that diagnosis and treatment may affect patients’ physical, mental, social and emotional health [14–22].

We aimed to study whether the quality of life (QoL) in testicular cancer survivors differs with time since cancer diagnosis, in other words, whether there are time-dependent effects of the disease and treatment on the age-adjusted QoL. We do not know if the quality of life changes with time after diagnosis, because there are no studies that measured QoL repeatedly over time. In this study we did not have longitudinal data, nevertheless, we administered the same questionnaire cross-sectionally to men diagnosed with testicular cancer from 2 to 10 years before. The study was carried out among the cases of the EPSAM case-control study and took advantage of the fact that in the EPSAM study patients diagnosed between 1997 and 2008 were all interviewed between 2008 and 2010, thus at different time distances from the diagnosis.

## Methods

### Study population

The study population has been previously described [23, 24]. Between 2008 and 2010, a population-based case-control study was conducted among testicular cancer patients diagnosed in the Province of Turin, Italy, between 1997 and 2008. Cases were all listed in the regional Hospital Discharge Registry (ICD-9 CM diagnostic code 186 for testicular cancer and ICD-9 CM surgical procedure code 623–624 for orchiectomy) and were contacted using two approaches. Patients followed at the University Hospital San Giovanni Battista (now named “Città della Scienza e della Salute di Torino”), the main hospital in the city of Turin, were contacted through their oncologist. The other patients were contacted through their general practitioners (345 GPs, 81% of the total number of contacted GPs, agreed to collaborate with the study). Consistently with a population-based approach, patients who were not residents in the Province of Turin were not included in the study. The same two sources were used to select the controls, who are however not included in this study that focuses on QoL among the cases. The study was approved by the local Ethical Committee and informed consent was obtained from each subject. The current study is thus a prevalence study on QoL conducted in 2008–2010 among all patients diagnosed with testicular cancer in the Province of Turin between 1997 and 2008. QoL was assessed at different time distances since diagnosis of testicular cancer: for 35 patients within 2 years since diagnosis, for 75 patients between 2 and 5 years since diagnosis and for 124 patients more than 5 years since diagnosis. Response rates were 49% among case-patients contacted through their GP, 82% among those contacted through their oncologist with an overall response rate of 57% and in total 274 study patients. We obtained a copy of the histological review for all cases, except one who was excluded from the study. Five cases who were not born in Italy were excluded from the study. Furthermore, following a complete-case approach, we excluded 25 cases who did not answer the questions related to QoL from the study questionnaire. Finally, we restricted the study to germ-cell testicular cancers, so 14 cases of the non-germ-cell origin or spermatocyte seminomas were excluded, resulting in 125 seminoma cases and 109 non-seminoma cases (mixed tumors were classified as nonseminomas). Thus, the study analyses included 234 cases. The EPSAM study was approved by the Ethical Committee (IRB) of the San Giovanni Battista Hospital–CTO/CRF/Maria Adelaide Hospital (Turin, Italy). Informed consent was obtained from all individual participants included in the study. All patients consented to the interviews in writing and the patients’ identities were protected by project id numbers in order to secure potential identification of the patients.

### Questionnaire data

QoL was assessed using the SF-12 (Short Form 12) generic health survey [25] that includes twelve questions. It produces two summary scores, PCS (Physical Composite Score) and MCS (Mental Composite Score), which evaluate the physical and mental health, respectively, ranging from 0 to 100, where 100 represents the highest level of QoL. SF-12 does not target a specific age or disease group and is a shorter and alternative form of the SF-36 (Short Form 36) [26].

### Statistical analyses

Since MCS and PCS in our study were not normally distributed, data were reported as median and inter-quantile range (IQR), and logistic quantile regression was used to estimate the association between time since diagnosis (considered both as categorical, <2, 2–5, 5+ years, and as a continuous variable with a 1-year scale) and QoL scores (modeling the median of the PCS and MCS [27, 28]). The calculated odds ratios reflect odds of being above the median for the exposed compared to the unexposed. The logistic quantile regression was adjusted for age at the compilation of the questionnaire, educational level (junior high school, high school and university degree), residence (city of Turin, the rest of the province of Turin) and method of contact of the case (oncologist or GP). Confidence intervals were obtained using bootstrap. Linearity on the logit scale was checked using restricted cubic splines for time since diagnosis and age at compilation.

Stratified analyses by histological type (seminoma/ non-seminoma) were also conducted. In order to evaluate the potential bias introduced by the low response proportion, we conducted a sensitivity analysis restricted to cases contacted through their oncologist because of the high response proportion (82%) in this group.

In addition, the QoL score means of the patients in our study were compared with those of the general Italian population, using previously published age-stratified (5 groups: <24, 25–34, 35–44, 45–54 and 55+ years of age) means of PCS and MCS [29, 30].

All analyses were conducted using STATA statistical software (version 13, College Station, TX: StataCorp LP).

## Results

Descriptive characteristics are reported in Table 1. Out of 234 patients, 78 (33.3%) were contacted through their oncologist and 156 (66.7%) were contacted through their GP. Regarding the histological type, 125 cases (53.4%) were seminomas and 109 cases (46.7%) were non-seminomas. The mean age at diagnosis was 34.5 years (aged between 16.4 and 57.3 years at diagnosis). The median time between diagnosis and compilation of the questionnaire was 5.3 years (IQR: 2.7–8.2); The median PCS score was 54.2 (IQR: 50.0–55.9) and the median MCS score was 50.2 (IQR: 40.9–55.1).

**Table 1:** Descriptive characteristics of the cases included into the study.

Characteristics	*N = 234*	*%*
**Year of birth**		
<1960	45	19.2
1960–1969	67	28.7
1970–1979	97	41.4
≥1980	25	10.7
**Age at diagnosis (years)**		
≤24	33	14.1
25–34	87	37.2
35–44	75	32
45+	39	16.7
**Age at compilation (years)**		
≤24	7	3
25–34	59	25.2
35–44	90	38.5
45–54	59	25.2
55+	19	8.1
**Method of contact**		
General practitioners	156	66.7
Hospital	78	33.3
**Residence**		
City of Turin	92	39.3
Rest of the province	142	60.7
**Histology type**		
Seminoma	125	53.4
Nonseminoma	109	46.6
**Educational level**		
Lower secondary school or less	85	36.3
Upper secondary school	95	40.6
University degree	54	23.1

S1 Table reports mean age-specific PCS and MCS scores for cases included in the study and general Italian population reported in the study by Kodraliu et al. [29]. The mean values of PCS and MCS calculated in the subjects of our study are consistent with age-specific scores among men of the general Italian population.

Table 2 reports the median and inter-quartile ranges of PCS and MCS, according to different characteristics of the testicular cancer patients. After adjustment by age, none of the variables was associated with median MCS, while age at diagnosis, educational level and histological type were associated with PCS.

**Table 2:** Physical and mental component scores distribution by cases’ characteristics.

Cases			Median (IQR)	
Characteristics	N = 234	%	PCS12 (IQR)	MCS12 (IQR)
**Overall score**			54.18 (50.00–55.91)	50.24 (40.95–55.13)
**1. Age at diagnosis** (mean age: 34.5)				
≤24	33	14.1	55.70 (54.42–56.82)	48.04 (37.54–55.13)
25–34	87	37.2	54.21 (50.38–56.00)	50.67 (41.30–53.39)
35–44	75	32	53.54 (48.80–55.88)	49.94 (41.86–55.87)
45+	39	16.7	53.55 (44.86–54.92)	49.98 (29.92–57.92)
p value^			0.04	0.51
**2. Age at compilation** (mean age: 40.2)				
≤24	7	3	55.91 (54.74–57.26)	52.98 (31.83–55.87)
25–34	59	25.2	54.18 (51.03–55.91)	50.60 (44.21–54.68)
35–44	90	38.5	54.32 (51.50–56.02)	50.17 (41.86–55.10)
45–54	59	25.2	52.81 (47.97–55.24)	50.85 (36.46–55.87)
55+	19	8.1	53.55 (41.32–55.40)	47.37 (29.43–55.96)
p value^			0.30	0.67
**3. Method of identification and contact**				
General practitioners	156	66.7	54.09 (50.52–55.91)	50.11 (40.60–55.09)
Hospital	78	33.3	54.14 (46.20–55.53)	50.67 (41.86–55.77)
p value^			0.90	0.72
adjusted p value*			0.57	0.88
**4. Histology type**				
Seminoma	125	53.4	54.18 (51.03–55.91)	50.24 (41.86–55.87)
Nonseminoma	109	46.6	54.07 (48.38–55.89)	50.58 (40.86–53.95)
p value^			0.74	0.74
adjusted p value*			0.03	0.84
**5. Education level**				
Lower secondary school or less	85	36.3	53.12 (46.65–54.31)	50.67 (40.25–55.87)
Upper secondary school	95	40.6	54.31 (50.51–56.44)	49.90 (37.54–54.32)
University degree	54	23.1	55.12 (52.83–55.91)	50.51 (43.06–56.87)
p value^			0.08	0.58
adjusted p value*			0.003	0.86
**6. Residence**				
City of Turin	92	39.3	54.19 (51.21–55.74)	50.34 (40.91–55.87)
Province of Turin	142	60.7	53.91(49.19–55.91)	50.29 (41.30–54.63)
p value^			0.58	0.88
adjusted p value*			0.88	0.84
**7. Time between diagnosis and compilation of the questionnarie**				
<2	35	15.3	54.18 (45.92–55.91)	51.14 (44.74–55.87)
2–5	75	31.9	53.82 (51.03–55.41)	49.94 (42.61–53.95)
5+	124	52.8	54.13 (50.19–55.91)	49.98 (37.26–55.50)
p value^			0.98	0.65
adjusted p value*			0.75	0.74

^Wald test for composite linear hypothesis after applying univariable quantile logistic regression.

* Adjusted for age at compilation.

Table 3 reports the estimated odds ratios of the median score in each category of time since diagnosis vs the reference (<2 years) when time is considered as a categorical, and for one year increase of time since cancer diagnosis when it is considered as a continuous variable. For example, 1.11 represents the adjusted odds ratio of the median score when the time since diagnosis is larger than 5 years compared to the reference (<2 years), where the odds are defined using the score instead of a probability, (PCS12-22.37)/(62.36-PCS12).

**Table 3:** ORs (odds ratios) and 95% confidence intervals (95% CI), estimated using logistic quantile regression, of median of PCS and MCS for time since cancer diagnosis.

Years since diagnosis	Cases, N = 234	PCS (Physical Composite Score)		MCS (Mental Composite Score)	
Categories	N (%)	Unadjusted	Adjusted *	Unadjusted	Adjusted *
		(50 quantiles)	(50 quantiles)	(50 quantiles)	(50 quantiles)
<2	36(15.3)	ref.	ref.	ref.	ref.
2–5	75(31.9)	0.95 (0.70–1.31), p = 0.74	0.95 (0.69–1.31), p = 0.77	0.88 (0.50–1.54), p = 0.65	0.76 (0.42–1.38), p = 0.37
5+	124(52.8)	0.98 (0.72–1.34), p = 0.91	1.11 (0.82–1.51), p = 0.49	0.86 (0.51–1.45), p = 0.57	0.71 (0.40–1.25), p = 0.23
1 year increase		0.99 (0.97–1.02), p = 0.83	1.01 (0.97–1.04), p = 0.59	0.97 (0.92–1.02), p = 0.29	0.96 (0.90–1.02), p = 0.18

* ORs (odds ratios) are adjusted for: age at compilation, educational level, residence and method of identification/contact.

**Fig 1:**
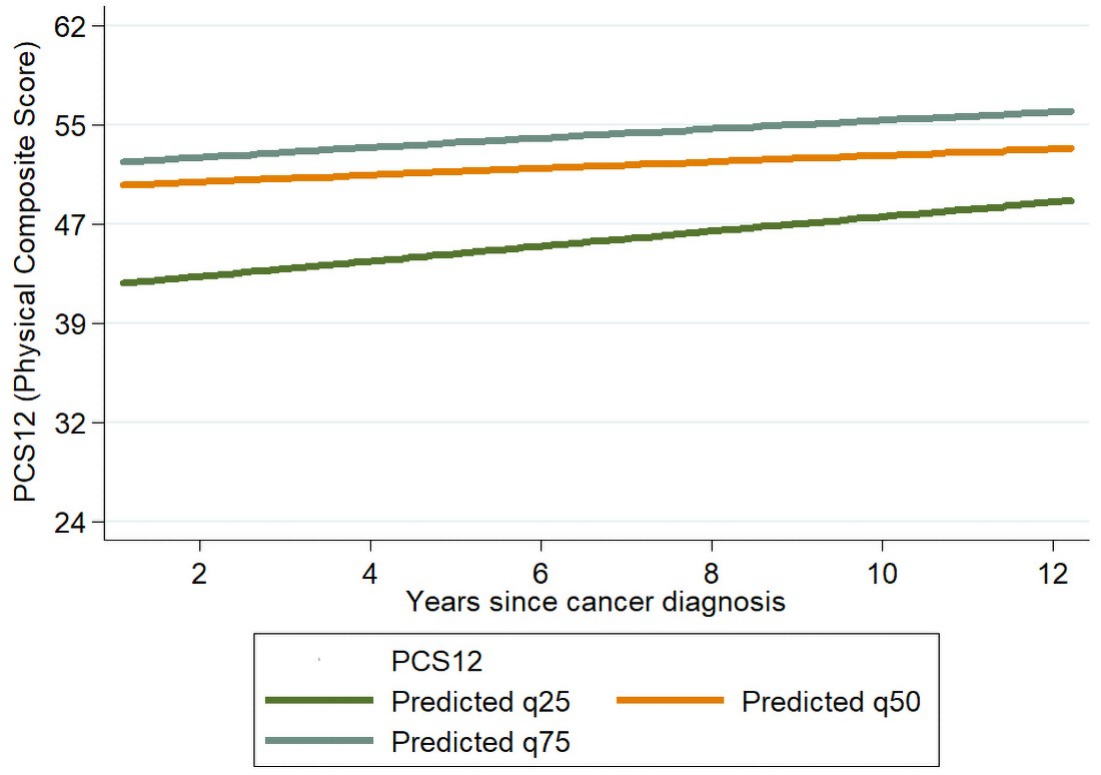
Scatterplot and line plot of predicted response for the 25^th^, 50^th^ and 75th quantile for PCS in nonseminomas cases, on time between diagnosis and compilation of the questionnaire. The lines refer to a subject with all categorical variables set at the reference values and aged 40 years at compilation of the questionnaire.

**Fig 2:**
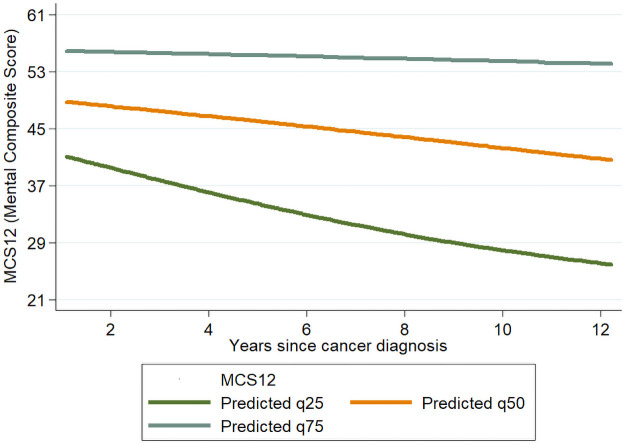
Scatterplot and line plot of predicted response for the 25^th^, 50^th^ and 75th quantile for MCS in nonseminomas cases, on time between diagnosis and compilation of the questionnaire. The lines refer to a subject with all categorical variables set at the reference values and aged 40 years at compilation of the questionnaire.

Table 4 reports the results stratified by histological type. Possible decline tendency of MCS over time since diagnosis, can be noticed in nonseminomas (adjusted OR for 1-year increase: 0.92, 95% CI:0.82–1.05, p = 0.23), for which there was also a positive association between time since diagnosis and PCS (for 5+ vs < 2 years 1.78, 95% CI: 1.17–2.73, p = 0.008; adjusted OR for 1 year increase: 1.03, 95% CI:0.98–1.08, p = 0.17). Figs 1 and 2 show the predicted PCS and MCS scores for the 25^th^, 50^th^ and 75^th^ quantiles by time since diagnosis in non-seminomas cases. Sensitivity analyses revealed a limited impact of non-response on our results. When analyses were restricted to the 78 cases contacted through their oncologist, results were consistent with those reported in Table 3 for the full cohort (PCS: adjusted OR: 1.06; MCS: adjusted OR: 1.00, for each 1-year increase in the time since diagnosis when considered as continuous).

**Table 4:** ORs (odds ratios) and 95% confidence intervals (95% CI), estimated using logistic quantile regression of median of PCS and MCS for time since cancer diagnosis of nonseminoma and seminoma cases.

Years since diagnosis	Nonseminomas cases, N = 109	PCS (Physical Composite Score)		MCS (Mental Composite Score)	
Categories	N (%)	Unadjusted	Adjusted*	Unadjusted	Adjusted*
		(50 quantiles)	(50 quantiles)	(50 quantiles)	(50 quantiles)
<2	19 (17.4)	ref.	ref.	ref.	ref.
2–5	33 (30.3)	1.21 (0.66–2.20), p = 0.53	1.29 (0.83–2.04), p = 0.25	0.70 (0.26–1.90), p = 0.48	0.74 (0.28–1.96), p = 0.55
5+	57 (52.3)	1.41 (0.81–2.44), p = 0.22	1.78 (1.17–2.73), p = 0.008	0.52 (0.21–1.28), p = 0.15	0.62 (0.25–1.54), p = 0.30
1 year increase		1.02 (0.96–1.09), p = 0.50	1.03 (0.98–1.08), p = 0.17	0.90 (0.82–1.00), p = 0.04	0.92 (0.82–1.05), p = 0.23
Years after diagnosis	Seminomas cases, N = 125	PCS (Physical Composite Score)		MCS (Mental Composite Score)	
Categories	N (%)	Unadjusted	Adjusted*	Unadjusted	Adjusted
		(50 quantiles)	(50 quantiles)	(50 quantiles)	(50 quantiles)
<2	16 (12.8)	ref.	ref.	ref.	ref.
2–5	42 (33.6)	1.04 (0.70–1.54), p = 0.83	0.90 (0.53–1.51), p = 0.67	0.89 (0.37–2.13), p = 0.79	0.79 (0.29–2.16), p = 0.65
5+	67 (53.6)	0.92 (0.63–1.33), p = 0.64	0.81 (0.48–1.38), p = 0.44	1.08 (0.47–2.47), p = 0.84	0.96 (0.35–2.63), p = 0.93
1 year increase		0.99 (0.95–1.02), p = 0.47	0.97 (0.92–1.03), p = 0.31	1.01 (0.94–1.09), p = 0.80	1.01 (0.91–1.12), p = 0.82

* ORs (odds ratios) are adjusted for: age at compilation, educational level, residence and method of identification/contact.

## Discussion

In a cross-sectional study of testicular cancer patients followed up for 1 to more than 10 years, we did not find clear evidence of an association between time since cancer diagnosis and patients’ QoL when we considered all germ-cell testicular cancer patients together. When the analyses were stratified by histology type, we found some evidence of a possible increase in PCS among nonseminomas. Although without clear statistical evidence, MCS among nonseminomas seems to be decreasing over time since cancer diagnosis.

A possible difference in changes in PCS and MCS over time by histology is difficult to explain. It could be due to the ages at which nonseminomas and seminomas are diagnosed (nonseminomas peak at around 25–30 years of age and seminomas at around 35 years of age) and because of the difference in the therapeutic approach between the two histology groups. Considering the different age peaks of testicular cancer, possible explanations for the mental health decreasing in non-seminomas cases, may be explained by the fact that problems with infertility and sexuality in testicular cancer survivors will be more discouraging for younger instead of older men. Besides, the literature supports that younger cancer survivors show greater anxiety and poorer QoL [31].

Quality of life among testicular cancer survivors has been a matter of interest for a long time and to our knowledge, there are no previous studies that assessed the relationship between time since testicular cancer diagnosis and patients’ QoL. Findings in our study, regarding mental and physical health, are consistent with the scientific knowledge up to now. Literature reviews [32–35] have shown that although testicular cancer survivors do not have serious disruptions to general health, however, they are more likely to experience mental health impairment as fatigue, anxiety, sexual problems and fear of cancer recurrence.

Other studies [14, 22, 36] that assessed the QoL among testicular cancer survivors concluded that QoL did not differ when compared with the age-matched general male population, except for slightly lower scores for mental health. Finally, two case-control studies have found that overall health status did not differ dramatically between cases and age-matched healthy male controls from the general population, but they did find that patients who received chemotherapy have lower PCS and MCS scores than those who did not [16, 17].

However, it is challenging to set in a context our results and make a comparison with previous research evidence. Previous studies have mainly used a wide range of different questionnaires to investigate the impact of chemotherapy, radiotherapy and acquired comorbidities on patients’ QoL. Moreover, we aimed to assess specifically the effect of time since cancer diagnosis.

The main two strengths of our study are: (i) its population-based design, (ii) the ability to analyze time since cancer diagnosis using a validated generic health survey. In a large study carried out to validate the SF-12 health survey in nine countries [30], SF-12 was proved to be a practical alternative to the SF-36 survey, for purposes in which the focus is on overall physical and mental health outcomes and when QoL is assessed together with several other research hypotheses, like in the EPSAM study.

Our study had also some limitations. First, it has a cross-sectional and not a longitudinal design, but it is still more informative, in terms of the change of QoL over time since diagnosis than studies measuring QoL at the same follow-up time for all patients. Second, our approach did not provide direct information on whether on average (irrespectively of time since diagnosis) the QoL of testicular cancer patients is higher or lower than the general population. In other words, our study does not test whether testicular cancer patients have a decreased QoL, but only if their QoL changes with time since diagnosis. For the purpose of clarifying and better explain our results, we compared QoL scores of our study cases with those of the general Italian population. The age-specific PCS in our testicular cancer patients did not differ from those of the general Italian population, but they had had a lower age-specific MCS. Furthermore, we faced a low response proportion (50%). A low response proportion is not uncommon in QoL studies, but nevertheless, it can introduce severe bias if participation depends on the quality of life of the patients. However, in a sensitivity analysis restricted to the patients contacted through their oncologist, we found little evidence of an impact of non-response on our study estimates.

Finally, we did not have access to the clinical charts of all testicular cancer patients so we could not reconstruct the treatment and co-morbidity history of the patients in the years between diagnosis and the participation in the study (this information was not asked in the study questionnaire, as the study had mainly the aim of identifying risk factors for the occurrence of testicular cancer). It should be noted however that post-diagnostic co-morbidity status and treatment are not confounders in our analysis but would have been rather mediators/explanators for our findings.

## Conclusions

Taking into account all the features mentioned above, to our knowledge, this is the first study specifically studying time since cancer diagnosis in association with the QoL of testicular cancer patients. Our main findings suggest that QoL in terms of physical and mental scores did not vary with time since diagnosis of testicular cancer, but may increase in PCS among nonseminomas.

## Supporting information

S1 Table Age specific normative scores and age specific sample scores for PCS and MCS. (DOC)

S1 Dataset EPSAM study, minimal data set. (XLS)

## Abbreviations

CI: Confidence Interval
GPs: General Practitioners
IQR: Inter-Quantile range
MCS: Mental Composite Score
OR: Odds Ratio
PCS: Physical Composite Score
QoL: Quality of Life
SF-12: Short Form 12
SF-36: Short Form 36
