# Supplementary material for: Quality of life among germ-cell testicular cancer survivors: The effect of time since cancer diagnosis
Source: PLoS One. 2021 Oct 6;16(10):e0258257. doi: 10.1371/journal.pone.0258257 (PMC8494333; doi:10.1371/journal.pone.0258257)
Supplement: S1 Table — (DOC) [file pone.0258257.s001.doc]

# Supporting information:

**S1 Table. Age specific normative scores and age specific sample scores for PCS and MCS**

| Age groups | Cases, N=234 (%) | PCS MCS  (Physical Composite Score) (Mental Composite Score) | | | |
| --- | --- | --- | --- | --- | --- |
|  |  | Age specific cases scores | Age specific normative scores | Age specific cases scores | Age specific normative scores |
| <24 | 7 (3) | 55.3 | 55 | 45.3 | 48.2 |
| 25-34 | 59 (25.2) | 51.1 | 54.3 | 47.3 | 49.2 |
| 35-44 | 90 (38.5) | 53.1 | 53.4 | 47.3 | 47.5 |
| 45-54 | 59 (25.2) | 50.0 | 52.2 | 46.9 | 47.5 |
| 55+ | 19 (8.1) | 48.4 | 49.5 | 43.2 | 45.7 |
